# Lightweight blockchain facilitated BIM data management for smart city operation and maintenance

**DOI:** 10.1371/journal.pone.0346900

**Published:** 2026-04-10

**Authors:** Keyu Chen, Zushun Li, Beiyu You, Xingyu Tao

**Affiliations:** 1 School of Civil Engineering and Architecture, Hainan University, Haikou, China; 2 Department of Building and Real Estate, Hong Kong Polytechnic University, Hong Kong SAR, China; Longgang Otorhinolaryngology Hospital & Shenzhen Key Laboratory of Otorhinolaryngology, Shenzhen Institute of Otorhinolaryngology, CHINA

## Abstract

Building Information Modelling (BIM) project operation and maintenance information management in smart cities currently faces challenges like data redundancy, a large number of management and maintenance personnel, and complex processes. Blockchain technology, with its decentralized and non-tamperable characteristics, offers a potential solution to these challenges. However, its direct application is not a panacea, as its complex deployment and cumbersome approval processes can introduce new challenges. Therefore, this paper proposes a lightweight blockchain-based BIM project O&M information management framework. A Design Science Research (DSR) methodology was used to construct this framework, aiming to optimize the information management process and ensure its efficient application. First, a one-to-many mapping tool was developed to allow a single blockchain node to register multiple users, thus simplifying the deployment process and enabling a lightweight blockchain. An InterPlanetary File System (IPFS)-based tool was also built for generating digital fingerprints to enhance data security and traceability. Secondly, to deal with the complex BIM collaboration process, multiple types of smart contracts were designed and developed to automate the coordination and management of various interactions and transactions in BIM projects, thereby improving the efficiency and accuracy of the entire collaboration process. Finally, this study validates the effectiveness of the proposed BIM information management approach using Hyperledger Fabric. The validation results show that the proposed approach is not only technically feasible in the field of BIM information management, but also effectively addresses key storage and efficiency challenges prevalent in smart city BIM applications in terms of performance metrics, including response latency and data processing throughput.

## 1. Introduction

With the rapid advancement of information technology, digital innovations have become deeply embedded in economic and social development, markedly enhancing their quality and efficiency [1]. Key technologies, including cloud computing, the Internet of Things (IoT), and blockchain, are recognized as pivotal solutions for the establishment of smart cities. These technologies are being actively explored globally [2]; studies indicate that over 80% of U.S. cities have articulated smart city strategies [3], with similar strategic principles proposed in Europe and ambitious blueprints implemented in China [4,5]. However, the path to building smart cities is fraught with challenges, particularly the persistence of information rigidity and data silos which impede healthy development [6]. To achieve smart city development, it is imperative to dismantle these silos and facilitate seamless, secure information sharing.

BIM technology is integral to the development of smart cities, offering robust support for collaborative communication across various disciplines. BIM effectively addresses issues such as design alterations and provides comprehensive construction data and information, thereby simplifying construction projects [7]. This technology reduces design errors and repetitive tasks and enhances both project progress and efficiency [8]. By integrating and managing information throughout the entire lifecycle of a building (from design and construction to operation), BIM facilitates real-time updates and sharing of building data. This capability allows project participants to immediately assess the impact of design changes and the interactions among different disciplines, thus further boosting efficiency and project advancement. At the same time, BIM has limitations in interoperability, change barriers, and design verifiability [9]. The reliance on a centralized database as the key data source introduces potential vulnerabilities, including susceptibility to data manipulation [10]and tampering with BIM records and documents [11], which compromises the authenticity and security of the data [12]. Moreover, during the operational phase, project teams might encounter risks related to the loss of data traceability and authenticity [13]. A significant concern is the risk of a single point of failure, especially when a third party has extensive access to or control over the platform [14]. Such vulnerabilities can disrupt project schedules, lead to data loss, and escalate costs [15]. Given these challenges, it is imperative to optimize and enhance the application of BIM technology. This involves strengthening security measures to safeguard data integrity and exploring decentralized approaches to mitigate the risks associated with centralized control.

The integration of blockchain technology into construction engineering is in its nascent stages but offers robust solutions to these cybersecurity challenges [16]. Kifokeris and Tezel [17] highlighted the potential value of integrating blockchain with lean construction, while its decentralized consensus mechanism ensures a secure distributed ledger. Recent empirical studies have demonstrated this potential [18]. Tao, et al. [19] introduced a blockchain-based BIM version control system. Zheng, et al. [20] developed “bcBIM” for sharing model hash values. Parn and Edwards [21] utilized blockchain for transparent workflows. Liu, et al. [22] proposed an architecture for storing BIM data, and Erri Pradeep, et al. [23] explored systems for ensuring change traceability. Despite these promising applications, integrating blockchain into smart city BIM projects remains challenging. Existing blockchain platforms often fall short of supporting the unique, complex collaborative requirements of BIM systems effectively. Furthermore, current deployment and registration processes are cumbersome and error-prone, complicating widespread adoption. There is a notable lack of practical research on implementing blockchain frameworks that are both lightweight and capable of managing BIM data in smart city environments.

To address the challenges of integrating blockchain with BIM in smart city projects, this study adopts a design science research (DSR) approach to develop a lightweight and practical research framework. The framework outlines the overall workflow and introduces several key innovations proposed in this paper. One major contribution is a one-to-many mapping tool that streamlines the blockchain registration process. This tool substantially reduces deployment complexity, improves system scalability, and strengthens data security. Given the multidisciplinary nature of BIM collaboration—where professionals from different domains must coordinate continuously—the study further develops multiple types of smart contracts to support complex collaborative workflows. In BIM projects, designers from various specialties are required to execute different smart contract operations to maintain an orderly and synchronized design process. To support this need, we introduce a classification and management system for organizing smart contracts across professional domains. This system enables the creation, invocation, and management of specialized smart contracts tailored to discipline-specific requirements. By incorporating multi-type smart contracts within a structured management framework, the proposed approach simplifies collaborative design processes and provides an efficient, customizable mechanism for cross-departmental coordination. This enhancement facilitates more consistent, traceable, and effective BIM team workflows, thereby improving both the efficiency and accuracy of BIM project management.

The organization of this paper is structured as follows. Section 2 delves into the technical background, thoroughly reviewing existing literature to identify current research gaps and challenges in integrating blockchain technology with BIM for smart city projects. Section 3 details the development process of the proposed lightweight blockchain (LB) framework. Section 4 evaluates the feasibility of the proposed framework. This section includes a detailed performance assessment based on practical implementation and experimental results, illustrating the framework’s capabilities in real-world scenarios. Section 5 discusses the innovative aspects of the LB framework and the current limitations that need to be addressed.

## 2. Literature review

### 2.1 Application of BIM technology in smart city management

The application of BIM technology in the construction industry offers substantial benefits, including cost reduction, time savings, and enhanced collaboration [24]. As argued by Karim, et al. [25], BIM supports a wide array of functionalities such as information management, simulation, automated safety checks, and risk detection. Waqar, et al. [26] highlight that one of BIM’s significant advantages is its capability to precisely identify potential risks before construction begins or during the design phase. Moreover, BIM technology greatly enhances the efficiency of data storage and retrieval, optimizing various management activities throughout a building’s lifecycle. It facilitates visual presentations [27], clash detection [28], cost optimization [29], team collaboration and communication [30], safety and security measures, among many other functions [31]. These capabilities not only improve the quality and efficiency of construction projects but also drive the digital transformation and intelligent advancement of the construction industry. Despite its relatively recent introduction, BIM has been extensively adopted across various construction and manufacturing sectors over the past few decades [32,33]. BIM’s utility extends beyond the design and construction phases to encompass the entire lifecycle of a building, from planning and design through construction, operation, maintenance, and even demolition [34]. This comprehensive application of BIM brings transformative changes and enhancements to the industry. For instance, during the design phase, BIM enables parametric modeling, design clash detection, collaborative design, and overall structural safety assessments [35,36]. Honghong, et al. [37]demonstrated how BIM technology can accurately align each component with parameter-driven information, significantly reducing construction costs and risks while ensuring continual improvements in construction quality. In the realm of O&M, Scianna, et al. [38] emphasized the integration of Internet of Things (IoT) systems with BIM models through a database management system. This integration facilitates real-time interactions between the physical structure and its digital BIM representation. Specifically, data captured by sensors are transmitted to the database management system, which conducts risk assessments of the actual structure, thus ensuring the project’s safety and reliability.

Globally, nations are developing blueprints to transform both existing and emerging urban areas into smarter and more sustainable cities [39]. This movement towards smart cities represents a significant trend in contemporary urban development [40]. BIM technology plays a pivotal role in this transformation by enabling digital management of buildings, municipal facilities, and other assets. Through the use of BIM models, cities can achieve real-time monitoring, maintenance, and management of urban assets, thereby enhancing the efficiency and effectiveness of urban management [41]. One of the pioneers in applying BIM technology to smart city development is Wang [42], who focuses on creating intelligent city models by integrating sensors across various infrastructure networks and buildings. This approach facilitates the collection and analysis of data crucial for smart urban management. Similarly, Kim, et al. [43] highlight the potential of BIM as a decision-support tool that can significantly aid sustainable large-scale urbanization efforts. In Singapore, Ho and Rajabifard [44] have proposed an innovative framework for the application of BIM at the city level, specifically targeting the development of BIM-based strategies for urban land management. Their work emphasizes integrating BIM into broader urban planning and management processes to enhance city functionality and sustainability. Yamamura, et al. [45] have taken a comprehensive approach by studying the integration of Geographic Information System (GIS) and BIM technologies within a smart city energy planning framework. This integration aims to provide a holistic analysis that identifies optimal techniques for adjusting smart city infrastructure and informs policy strategies for sustainable urban development. Through these diverse applications, BIM technology not only supports the digital management of urban assets but also contributes to the strategic planning and implementation of smart city initiatives, driving the evolution of more efficient, sustainable, and intelligent urban environments.

However, in traditional centralized architectures, storing data in centralized servers or client-side systems poses a higher risk of data tampering and manipulation [46]. Centralized databases are prone to frequent server failures due to their inherent instability, which can severely disrupt the normal operations of the BIM platform. These disruptions not only jeopardize the continuity and efficiency of the project but also risk the loss or corruption of critical data. Such losses can have substantial adverse impacts on project timelines and overall productivity. A more pressing concern is the vulnerability of centralized BIM platforms to cybersecurity threats. Insufficient security measures can expose these platforms to hacker attacks, where malicious actors exploit security loopholes to steal or manipulate sensitive data. This unauthorized access can severely compromise project decision-making and implementation, leading to significant operational setbacks. Additionally, the threat of cyber-attacks such as viruses and Trojans poses a serious risk to the integrity of the BIM platform. These attacks can degrade system performance, leading to slowdowns or even complete system paralysis, which can incur immeasurable losses to the project. To address these challenges, the integration of blockchain technology offers a compelling solution. Blockchain’s decentralized architecture and robust security features can substantially enhance the efficiency, security, and transparency of BIM project information management. By leveraging blockchain’s ability to provide secure, tamper-proof records and its resistance to single points of failure, the resilience and reliability of BIM platforms can be significantly improved. This integration not only mitigates the risks associated with centralized data storage but also promotes more secure and transparent management of project information.

### 2.2 Blockchain-BIM integration

Blockchain technology represents a revolutionary approach to data management through its decentralized, distributed ledger system. This technology employs a unique blockchain structure that ensures tamper-proof and secure data storage, allowing each participant in the network to maintain a complete and immutable record set within their local database [47]. This inherent design guarantees data consistency and security across the network. Central to blockchain’s operation is its consensus mechanism, which establishes the governing rules for validating transactions and maintaining the integrity of the blockchain. This mechanism not only verifies the credentials of users but also determines the validity of each block before it is incorporated into the overall chain [48]. Once information is submitted and validated within the blockchain, it is rapidly propagated to all network nodes and permanently recorded, preventing unauthorized tampering and ensuring data integrity and legitimacy [49]. Moreover, blockchain facilitates secure communication and trust establishment within the network through its sophisticated encryption algorithms and consensus protocols. This technology eliminates the need for third-party intermediaries, enabling direct and secure transactions and information exchanges between individual nodes. The decentralized nature of blockchain technology makes it inherently resistant to single-point failures and network attacks, significantly enhancing the reliability and stability of the system. Additionally, because all transaction records on the blockchain are public and traceable, any participant can independently verify the authenticity and integrity of the data [50]. This transparency fosters trust among participants and encourages cooperation and collaboration, which is particularly beneficial for multi-party projects. Thus, blockchain technology provides robust support for ensuring secure, efficient, and reliable data management and communication in collaborative environments.

Recent research within the construction industry underscores the significant potential of blockchain technology, highlighting its role in driving digital transformation, enhancing collaboration efficiency, and fostering trust among stakeholders. The application of blockchain in construction shows great promise, prompting extensive exploration by researchers. Recent studies have demonstrated the feasibility of blockchain technology in driving digital transformation, improving collaboration efficiency, and enhancing trust within the construction sector [51]. Li, et al. [14]examined the challenges and opportunities of integrating blockchain into engineering processes, proposing a multidimensional conceptual model to optimize its application within the construction industry. Olawumi, et al. [52] surveyed various digital tools and technologies, focusing on blockchain’s potential to enable integrated construction projects. Lee, et al. [53] leveraged IoT and digital twin technologies within a blockchain system to capture construction information and facilitate information integration and simulation. Qian and Papadonikolaki [54] investigated trust dynamics within the construction supply chain, highlighting blockchain’s role in enhancing systematic trust and fostering stakeholder communication and cooperation. Majeed, et al. [55] explored the synergy between IoT-enabled smart cities and blockchain-based Distributed Ledger technology, aiming to create a more secure and decentralized data transfer process. Casino, et al. [56] argued for blockchain’s potential to drive building automation through the integration of design documentation and equipment records, enabling historical archival review and research development. These studies collectively demonstrate the diverse applications and benefits of blockchain technology within the construction industry, from enhancing collaboration and trust to driving innovation and efficiency. The research by Lawal and Nawari [57] demonstrates that blockchain-based City Information Modeling (CIM) integration can establish an efficient and secure platform for collaboration and data sharing, while also opening up new research opportunities for in-depth exploration of digital modeling and smart technologies.

The BIM process involves extensive data sharing and collaborative design efforts among various stakeholders, and the integration of blockchain technology with BIM holds significant promise for enhancing overall collaboration [58]. Celik, et al. [59] proposed a blockchain-based BIM data sourcing model that effectively improves the efficiency of BIM implementation, enhances data sharing confidence, reduces project costs, and enhances risk management capabilities. Many existing studies focus on storing BIM models in centralized databases while utilizing blockchain ledgers for sharing design changes and other attributes [60]. For instance, Dounas, et al. [61] adopted a strategy of storing modified objects rather than entire models. The integration of blockchain technology into BIM platforms has substantially increased the security and reliability of information storage and management, creating a more dependable environment for design collaboration within the construction engineering industry. Research by scholars such as Nawari and Ravindran [62] demonstrates that blockchain technology can provide more reliable data storage and refined permission management solutions in BIM workflows. As an integral component of the construction industry’s digital transformation, the BIM process continues to facilitate seamless data sharing among multiple parties and efficient collaborative design practices.

Despite the significant potential of blockchain technology in the construction and engineering sector, its implementation and application remain at an early stage, and various challenges and limitations have been encountered. The management of BIM data in smart cities involves complex multi-departmental and multi-disciplinary collaboration, necessitating a sufficient number of blockchain nodes to cater to diverse participant needs. However, research in this area is still lacking, particularly concerning the effective integration of resources from multiple stakeholders, enhancement of data management efficiency, assurance of data security, and facilitation of interdisciplinary collaboration. Furthermore, existing smart contracts often struggle to independently handle complex collaborative tasks, as they are typically designed for single, linear business logic rather than multifaceted and dynamically evolving projects. Additionally, previous studies have been deemed unsuitable for efficient smart city applications due to their cumbersome nature. Thus, this paper proposes an innovative fusion of LB technology with multiple types of smart contracts. At the core of this lightweight design is the simplification of the user registration process and optimization of node configuration, enabling all construction industry stakeholders to conveniently leverage blockchain technology. Simultaneously, the introduction of multiple types of smart contracts facilitates collaborative operations among different contracts, allowing them to collectively address complex task demands in construction projects. This integration further enhances the simulation and management efficiency of the dynamic collaborative environment.

## 3. Research methodology

**Fig 1:**
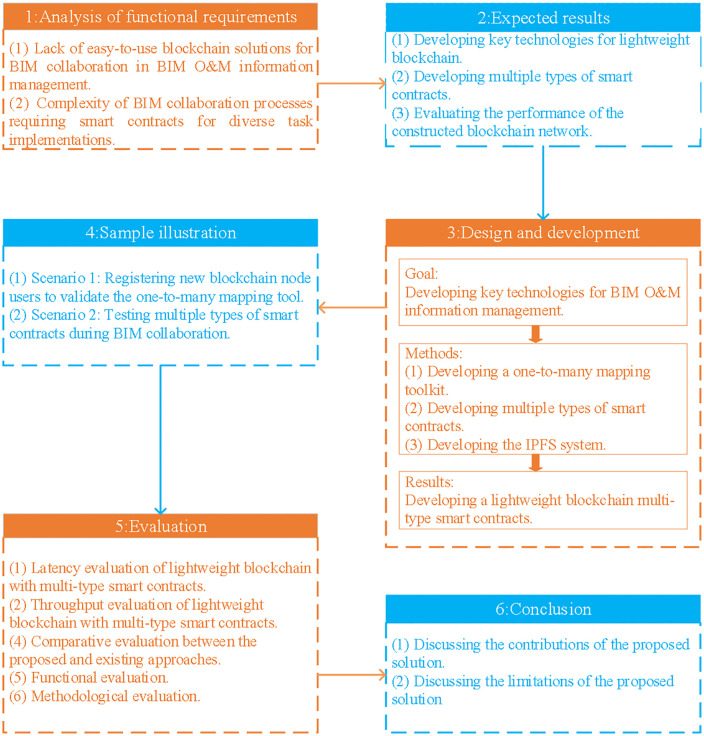
DSR research methodology step diagram.

This paper focuses on the development of a LB system integrated with multi-type smart contracts, guided by the principles of Design Science Research (DSR) [63]. The implementation process is meticulously designed to ensure a comprehensive and practical application of these technologies within the construction industry. As illustrated in Fig 1, the methodology encompasses six critical steps that collectively drive the development and deployment of the proposed system.

(1) Section 1 introduces the core research problem, identifying significant challenges in BIM projects related to the absence of user-friendly blockchain solutions and the intricate requirements for BIM collaboration. These challenges necessitate the use of complex multi-type smart contracts for the secure management of BIM information collaboration, resulting in design inefficiencies. This paper is motivated by the need to address these inefficiencies through a targeted research approach.
(2) The proposed solution entails the development of an LB system integrated with multiple types of smart contracts. This approach is designed to simplify deployment, enhance collaboration efficiency, and ensure secure BIM information management. The solution’s feasibility and effectiveness will be validated and evaluated throughout the study.
(3) This research adopts a multi-faceted methodology to develop robust technologies for BIM O&M information management. Initially, a one-to-many mapping toolkit is created to streamline the management and application of BIM data. Subsequently, diverse smart contracts are developed to augment the system’s flexibility and operability. Additionally, a distributed storage system based on IPFS is implemented to secure data and maintain traceability. These components collectively enable the construction of an LB and multi-type smart contract system that delivers efficient, secure, and reliable support for BIM O&M information management.
(4) The validation section exemplifies the practical application of the LB and multi-type smart contracts, demonstrating their efficacy in real-world scenarios. This section provides concrete evidence of how these technologies can be effectively applied to enhance BIM project management.
(5) The performance of the developed system is rigorously evaluated against key metrics, specifically latency and throughput, to ensure its capability to meet operational demands.
(6) The study concludes with a discussion of the advantages and limitations of the proposed solution. The findings, which contribute to the academic discourse on blockchain and BIM integration, will be disseminated through publication in an academic journal.

### 3.1 The analysis of information management needs

In building engineering, the design process typically begins with Computer-Aided Design (CAD) drawings, which are used to represent the initial design goals and anticipated outcomes. The transition from CAD to a BIM environment involves several specialized teams: the architectural team, the structural team, and the Mechanical, Electrical, and Plumbing (MEP) team. These teams collaborate to create a comprehensive BIM model based on the initial CAD drawings. The architectural team, in particular, often comprises multiple modelers who work on the same architectural model at different times. This staggered and distributed effort can introduce complexities in maintaining model coherence and consistency. Before the final model is delivered, it undergoes a thorough review by the management team. This review considers various aspects of the model and incorporates client feedback, which is communicated to the team leader for necessary adjustments. However, the collaborative process in BIM management is frequently challenged by the fragmented and diverse nature of these teams. Effective collaboration requires seamless integration of contributions from different disciplines, which often necessitates time-consuming file conversions and data integration efforts. These processes can lead to inefficiencies and increase the risk of data loss or inconsistencies. Addressing these issues is crucial for enhancing the overall efficiency and reliability of BIM management in building engineering.

**Fig 2:**
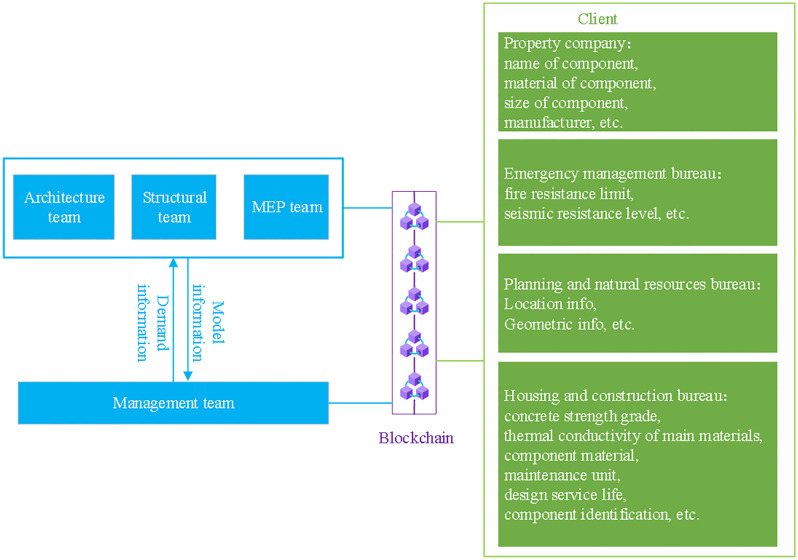
BIM information requirements.

The inherent complexity and multi-layered nature of building models pose significant challenges in information sharing among different teams involved in BIM projects. Often, each team is primarily focused on its specific section of the model, which can result in overlooking the design requirements and potential conflict points of other teams. This “information silo” effect hinders effective collaboration and leads to communication breakdowns, thereby compromising the overall accuracy and consistency of the building model. Moreover, the disparity in skill levels and experience among team members further exacerbates these issues. Some modelers may produce highly detailed and precise representations, while others might simplify the models excessively or omit critical construction details. This variability in modeling quality can lead to inconsistencies in the level of detail across different parts of the model, as shown in Fig 2, which illustrates the need for varying degrees of granularity for different administrative purposes. Additionally, architectural model review and revision processes are often fraught with challenges. Coordination and quality assurance become particularly cumbersome when team members are working asynchronously and from disparate locations. The absence of robust real-time collaboration platforms and workflows can cause significant delays and inefficiencies in the review process, further complicating the timely and accurate refinement of the model. These issues underscore the necessity for enhanced tools and methodologies to streamline communication and ensure consistency in BIM management practices.

BIM management is characterized by the extensive generation and management of diverse data, encompassing building components, attribute information, and material specifications. This data must be collaboratively shared and utilized by various teams for effective model analysis and development. However, the complexity and sheer volume of data present significant challenges for data sharing and synchronization. The absence of standardized and detailed BIM specifications, particularly in the context of smart cities, further complicates post-project modifications and adjustments. To address these challenges, teams ideally require a unified data management and collaboration platform capable of facilitating seamless data exchange and synchronization. Unfortunately, existing platforms often lack the maturity and flexibility needed to support the intricate and dynamic requirements of multi-disciplinary collaboration. Effective data management in BIM also necessitates rigorous version control and tracking mechanisms to maintain data consistency and ensure traceability throughout the project lifecycle. BIM management demands continuous interaction and coordination among various stakeholders, including architectural, structural, and MEP teams. Resolving model conflicts and issues typically requires frequent communication and negotiation among these groups. However, the involvement of numerous participants, often dispersed across different geographical locations, makes face-to-face meetings impractical. This geographic and logistical fragmentation impedes effective communication and negotiation, highlighting the need for advanced collaborative tools and strategies to facilitate seamless interaction in BIM environments.

The integration of blockchain technology into BIM collaboration presents a compelling solution due to its intrinsic attributes of high transparency, traceability, and the security provided by immutable digital signatures. Blockchain enables all stakeholders to access and verify real-time model information updates, ensuring data consistency and enhancing overall security. Effective participation in a blockchain network requires each team member to possess a foundational level of technical expertise and a commitment to resource investment for registration and ongoing involvement. Moreover, the complexity of collaborative tasks often necessitates the interaction and coordination of multiple smart contracts. These contracts must be designed to handle specific project requirements efficiently. Relying on a single smart contract is insufficient for managing the multifaceted and dynamic nature of modern construction projects. Therefore, it is imperative that smart contracts are meticulously crafted and strategically deployed to align with the unique demands of each project. This approach not only facilitates a more efficient and secure collaborative environment but also optimizes the performance and reliability of blockchain applications in BIM projects.

### 3.2 The development of LB

#### 3.2.1 Development principles.

**Fig 3:**
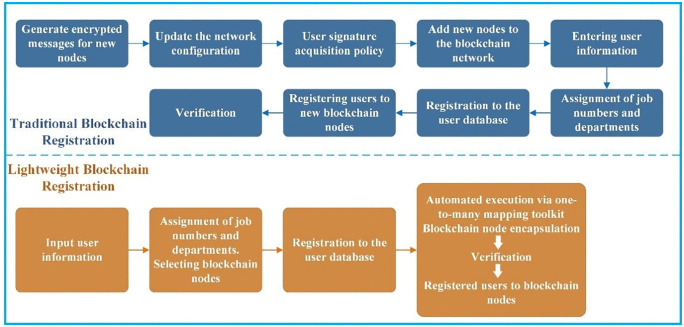
Flowchart of traditional blockchain registration vs. lightweight blockchain registration.

The integration of blockchain technology is crucial in advancing the O&M management of BIM projects within smart cities. Blockchain’s application in this domain goes beyond fulfilling the fundamental requirements of project O&M. It enhances and broadens the existing management systems across various dimensions. This technology’s transformative impact is guided by three core principles:

Principle 1: The implementation of LB technology significantly streamlines the user registration process, enhancing both the accessibility and efficiency of blockchain integration in smart city BIM projects. Traditional blockchain registration often requires users to navigate complex operational steps, leading to increased learning and time costs. To mitigate these challenges, this study introduces a one-to-many mapping toolkit that encapsulates the LB within a user-friendly service package. This toolkit simplifies the registration process, allowing users to complete their registration swiftly without additional configuration efforts. As illustrated in Fig 3, the streamlined registration process facilitated by the LB is markedly more concise and efficient compared to conventional blockchain registration methods.
Principle 2: The application of LB technology serves as an augmentation to, rather than a replacement for, existing BIM technologies in smart city projects. This approach preserves the foundational operational logic of BIM while integrating blockchain to enhance the security and reliability of data storage and transmission. By combining these technologies, we leverage the strengths of BIM and complement it with the robust data management capabilities of blockchain. This synergy not only enhances the integrity and reliability of BIM data but also creates a more secure environment for the management and flow of construction information.
Principle 3: In collaborative design processes, the integrity and authenticity of BIM data are paramount. This study addresses these needs by automatically generating hash values for shared BIM data, which are subsequently stored in the blockchain. The hash value acts as a unique digital fingerprint, providing a singular identifier for the BIM data content. Any tampering with the data results in a change in its hash value, thus allowing for immediate detection of unauthorized modifications. By storing these hash values on the blockchain, we ensure long-term integrity and authenticity of the BIM data, while also enhancing its transparency and traceability. This method provides robust data support for the operation and maintenance management of smart city projects, safeguarding against data tampering and ensuring reliable data provenance.

#### 3.2.2 Development of key technologies.

**Fig 4:**
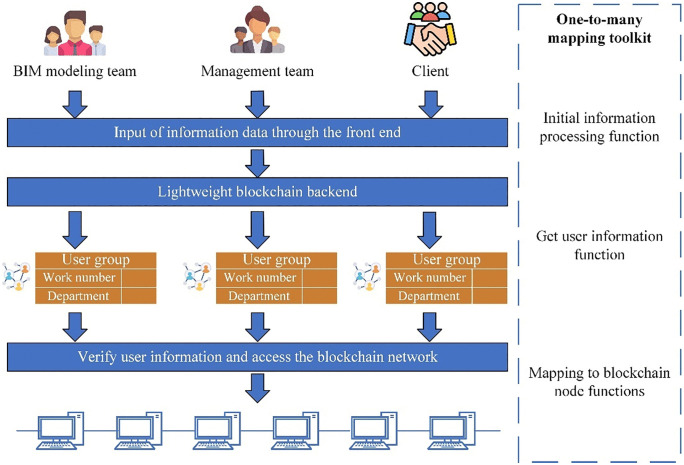
One-to-many mapping toolkit framework.

The primary aim in developing the one-to-many mapping toolkit is to achieve LB functionality, facilitating shared node utilization by multiple users, as depicted in Fig 4. This design philosophy targets a reduction in user threshold and operational complexity associated with blockchain adoption. The toolkit encompasses four primary functions.

**Fig 5:**
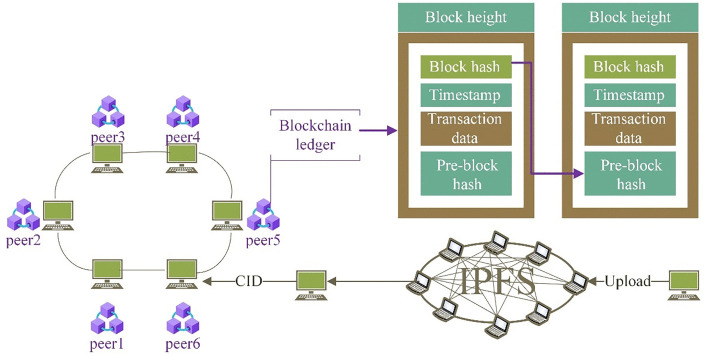
Schematic of IPFS integration with blockchain.

The Initial Information Processing function serves as the foundational element of the toolkit, initiating BIM file processing by interfacing with the IPFS. Fig 5 illustrates the integration of the IPFS system with blockchain technology. IPFS, a distributed file storage system, ensures file integrity and permanence through content addressing and version control mechanisms [64]. Within the one-to-many mapping toolkit, IPFS functions as the platform for processing and storing BIM files, which contain vital information for building projects. Leveraging the distributed storage and decentralized architecture of IPFS, multiple users can securely share access and operational privileges for the same BIM file. Upon completing the initialization process of BIM files, IPFS generates a unique string of hash values known as CID. CID uniquely identifies the stored file or data block within IPFS, facilitating precise data retrieval and verification, thereby ensuring data integrity and trustworthiness. Another critical component of the toolkit is the User Information Capture function, which collects essential user details such as work number and professional team affiliation. This information plays a pivotal role in subsequent transaction encapsulation and authentication processes. The Transaction Encapsulation function, a core aspect of the toolkit, integrates user information with initial data to form complete transaction data packets. These packets, considered pending transactions, await encapsulation into blockchain blocks. This ensures that each transaction packet includes accurate user and initial information, thus validating the transaction’s legitimacy and integrity.

Before transmitting transaction packets to the blockchain network, the Mapping to Blockchain Node feature performs a validation step on the user’s work number. This step is crucial for connecting users to the appropriate blockchain node service. By validating either the department name or work number, the one-to-many mapping toolkit guarantees that each user accesses only the designated blockchain node, mitigating data clutter and permission-related issues. This feature capitalizes on the decentralized and distributed nature of blockchain technology to seamlessly match users with specific blockchain nodes. The precise mapping mechanism not only upholds data integrity and privacy but also enhances transaction efficiency and security within the system.

In practice, the user-to-node mapping is implemented via a lightweight registry maintained by the consortium administrator. Each user is represented by a unique identifier (e.g., work number) together with organizational metadata (e.g., department/team), and is associated with a designated node endpoint. During access, the toolkit validates the user identifier and then performs a deterministic lookup (work number/department to node endpoint) to route all requests to the assigned node service. This design prevents users from arbitrarily switching endpoints to access other nodes, and therefore enforces node-level access separation while keeping the onboarding procedure lightweight. In combination with the role checks in smart contracts (Section 3.3.1), it forms a two-level permission model for collaborative BIM O&M.

### 3.3 Multi-type smart contract framework

A smart contract, a form of executable code deployed within the blockchain network, automatically executes a predefined program upon meeting specific conditions. It serves as an indispensable tool for facilitating blockchain-assisted BIM collaboration, offering rapid execution at the millisecond level. In the process, when a modeler deploys a smart contract into the blockchain network and submits a model-related query via the frontend interface, a transaction containing the model’s hash fingerprint and timestamp is transmitted to the smart contract. Subsequently, an endorsement node validates the transaction’s legitimacy. Once verified, the smart contract forwards the transaction to a sorting node, responsible for chronologically bundling the transaction into a new block.

#### 3.3.1 Multi-type smart contract mechanisms.

**Fig 6:**
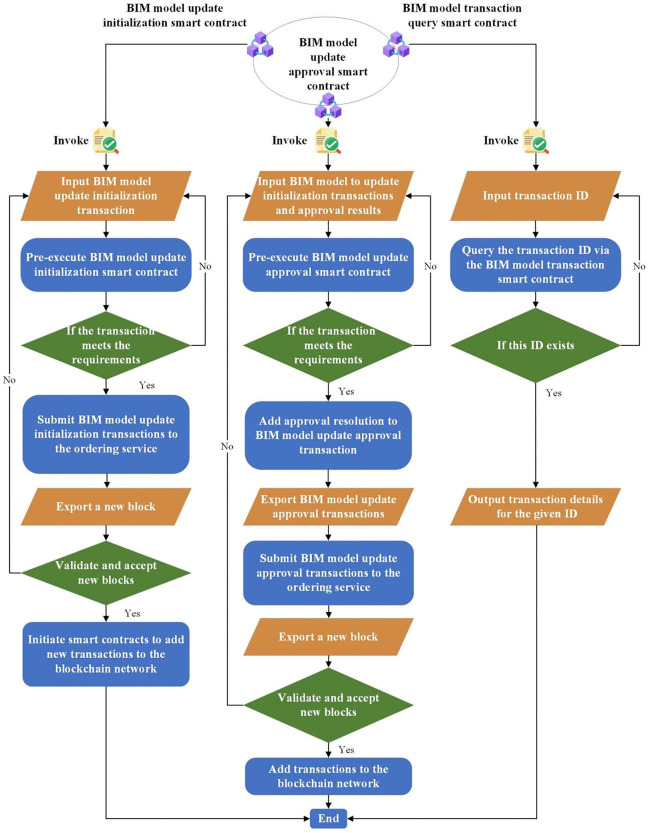
Multi-type smart contract mechanisms.

In this section, we present the development and functionality of three smart contracts tailored for BIM model management: the BIM model update initialization smart contract, the BIM model update approval smart contract, and the BIM model transaction query smart contract. The algorithms for each smart contract are depicted in Fig 6. To clarify the structure and execution logic, each smart contract takes a transaction request and the invoker identity as inputs, performs legitimacy validation (e.g., identity/signature and transaction integrity), and then executes a contract-specific state transition to generate the corresponding on-chain record or query result. Specifically, the initialization contract encapsulates validated update metadata into a new block for consensus dissemination, the approval contract records approval status/proof by generating a traceable approval transaction, and the query contract supports historical block tracing and returns detailed records to authorized users. The BIM model update initialization smart contract first validates transactions to ensure their legitimacy and accuracy. Upon validation, it retrieves the endorser’s signature from the blockchain and incorporates it into the transaction information. Subsequently, all pertinent data is encapsulated into a new block and disseminated across the network via a consensus algorithm, ensuring transaction transparency and security. Each design member verifies the received block’s legitimacy before its inclusion in the chain, thereby upholding the system’s trustworthiness and stability. Similarly, the BIM model update approval smart contract follows a validation protocol, appending update approvals and proof of approval functionality to validated transactions, thereby generating new transactions. This approach enhances transaction transparency and system security by clearly documenting approval status and providing additional validation. The BIM model transaction query smart contract facilitates historical data block tracking, enabling designers to efficiently trace previous transaction records and access detailed historical data. This tracking mechanism supports project management by providing insights into the BIM model’s evolution, aiding informed decision-making. User roles and permissions are clearly defined within the system, with modelers authorized to utilize the BIM model update initialization and transaction query smart contracts. This authorization empowers modelers to manage BIM models effectively, facilitating quick access to project-critical information. Professional leaders possess authorization for all three smart contracts, underscoring their pivotal role in BIM model review. The BIM model update approval smart contract acts as an auditing tool, ensuring a thorough review of model updates. Models passing review undergo a release process, with final approval vested in the BIM general manager, safeguarding data integrity and project quality. To explicitly enforce these permissions, the smart contracts implement role-based access control (RBAC) at the function level. The invoker identity (e.g., blockchain account/certificate) is checked against the pre-defined role assignment before executing any state update. Concretely, only authorized modelers can invoke update initialization and transaction query functions, while only professional leaders (and the BIM general manager for final release) can invoke approval-related functions; any unauthorized invocation is rejected prior to block generation. This contract-level enforcement remains effective even if a user can reach a node endpoint, thereby providing strict permission control for collaborative operations.

The operational design meticulously considers each department’s functional responsibilities, facilitating scientific and standardized information management. Clear division of labor between modelers and department heads ensures BIM model updates and streamlined approval processes. The utilization of smart contracts significantly simplifies procedures, enhancing operational efficiency and accuracy. This operational framework not only safeguards BIM model data quality but also augments system transparency. The synergistic execution of tasks by smart contracts within each department fosters an efficient information management network. This network facilitates tracking of BIM model update history and provides team members with convenient query capabilities, streamlining workflows and bolstering efficiency.

#### 3.3.2 Development of multiple types of smart contracts.

Maintaining stability in file names is paramount in the functionality of the initial update smart contract for BIM models. For instance, adhering to the specifications outlined in the Shenzhen existing important buildings modeling delivery technical guidelines, where file names remain unchanged throughout the design process, enables relevant designers to effectively trace the BIM model’s update history on the blockchain using the file name. These consistent naming conventions, exemplified by formats like 1#_A_F1_jx_V1.0, serve as dependable retrieval identifiers supporting the comprehensive history tracking of BIM models.

**Fig 7:**
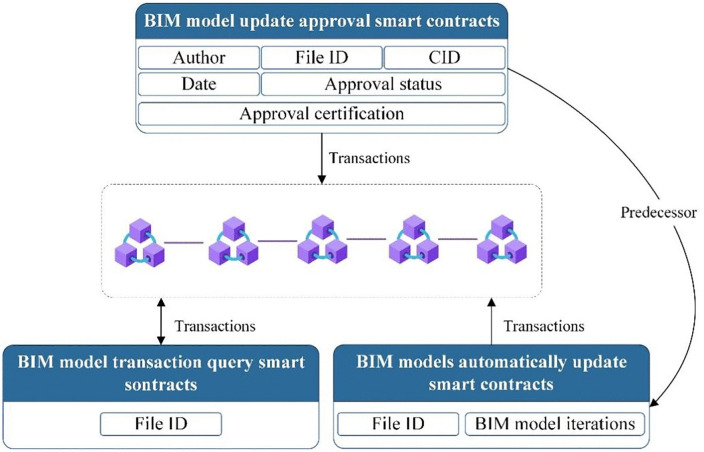
Workflow of the multi-type smart contract.

Designers can seamlessly access vital BIM information through CID utilization, facilitating direct access to BIM files stored in the IPFS. CIDs serve as unique identifiers, ensuring precise file location within IPFS, streamlining BIM model file storage and updates while upholding data integrity and security. The operational workflow of multi-type smart contracts, illustrated in Fig 7, further refines the BIM update approval process. Leveraging data obtained from the BIM model’s initial update smart contract, the approval system comprehensively covers all model aspects. Concurrently, the introduction of Approval Status and Proof of Approval functions enhances transparency and credibility. The pivotal role of approval status lies in visualizing the final decision of the responsible party, offering team members a clear understanding of each BIM model’s current approval status for streamlined workflow organization and collaboration. Augmenting credibility, the Proof of Approval feature mandates authorized user involvement in publishing BIM model updates through approved smart contracts. Notably, the blockchain database design, comprising chained and global databases, ensures data comparability and efficient global status management. Smart contract updates and approvals are exclusively recorded in the chained database, preserving system stability and efficiency. This design isolates transactional activities, enabling efficient global database query performance. The system’s capability to automatically track BIM model evolution through update recording enhances designer query convenience and accuracy, fostering workflow efficiency and reducing human error likelihood. Lastly, the BIM model transaction query smart contract introduction enriches system functionality, empowering designers to swiftly retrieve transaction information by querying BIM file names. This flexible query mechanism facilitates expedited access to critical information, fostering seamless project progression.

## 4. Sample verification

Blockchain technology remains underutilized in construction engineering. This section uses a smart city BIM project scenario-based approach to demonstrate the applicability and performance of the research model. Scenario 1 assesses the efficacy of the developed LB in simplifying user registration processes, while Scenario 2 evaluates the capability of multi-type smart contracts in facilitating BIM collaboration. To ensure the computational rigor of the proposed methodology, quantitative analysis is conducted. Latency and throughput serve as primary metrics, gauging the performance of the research methodology. All reported latency/throughput results are obtained using Hyperledger Caliper under the same network configuration, and each experiment is repeated for 30 runs; we report the averaged values across runs.

### 4.1 Validation environment preparation

The research utilized a test environment for smart city BIM projects with the following components: (1) VMware 17 virtual machine, (2) Ubuntu 22.04 with 8GB of RAM and 130GB of disk space, (3) six VM containers to isolate different blockchain peers, (4) IPFS version 0.5.0 and Hyperledger Fabric version 2.4.0 installed to establish the blockchain network, and (5) Hyperledger Explorer configured for transaction detail visualization and Caliper configured to evaluate the latency and throughput of the constructed blockchain network. Caliper generates controlled transaction and query workloads and reports end-to-end latency and throughput, while Hyperledger Explorer is used to inspect on-chain transaction records during the experiments. In this testbed, the consortium blockchain network consists of six peer nodes, each isolated in a separate VM container, and the same node configuration is used throughout the evaluation.

### 4.2 Validation scenarios

**Fig 8:**
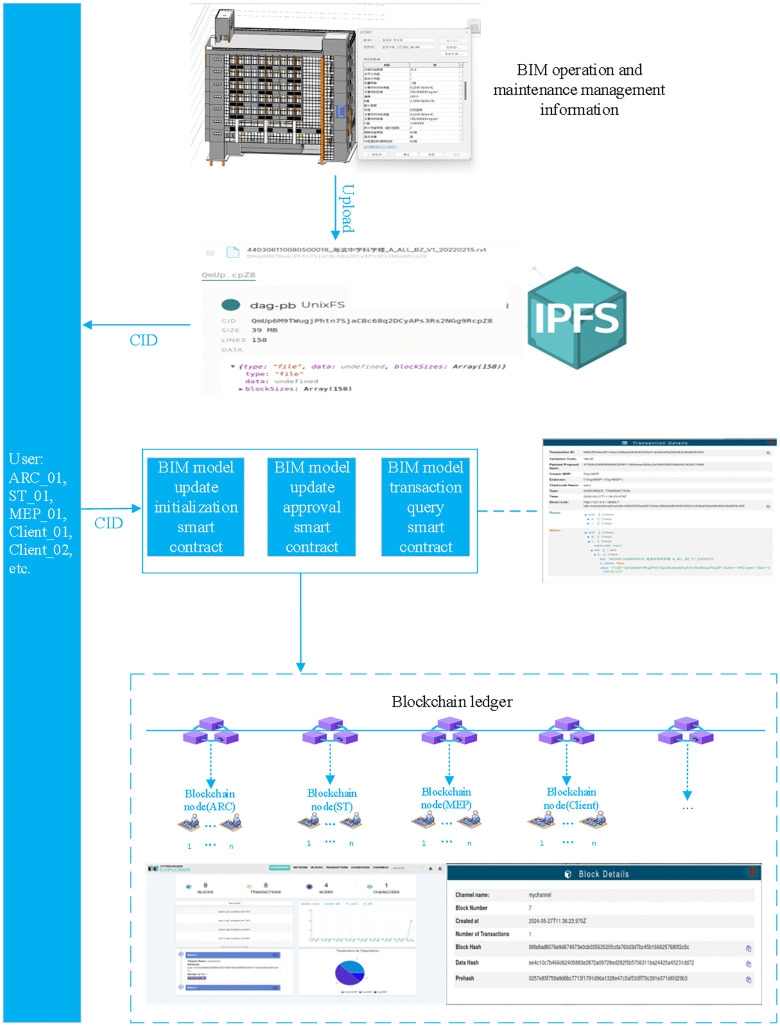
Schematic diagram of the verification scenario.

In this study, we selected the information management of a smart city BIM project in Shenzhen as a case study. The schematic diagram of the verification scenario is shown in Fig 8. The process begins with the administrator initiating user registration and assigning unique identifiers, such as ARC_01, ST_01, and MEP_01. Utilizing a one-to-many mapping toolkit, users seamlessly connect to blockchain nodes and invoke smart contracts to share BIM operational information, accompanied by corresponding CIDs from IPFS. Stakeholders such as emergency management departments and the housing and urban-rural development bureau verify the newly uploaded information on the blockchain to ensure transaction integrity. BIM construction modelers update smart contracts embedded with CIDs using BIM information, which are then verified based on government-specified parameters. Upon approval, the updated information and approval certificates are propagated across all nodes. The smart contract for querying BIM information transactions allows authorized professional departments and users to access BIM metadata and transaction details. This operational information management system enhances the transparency and traceability of BIM data, provides real-time access for emergency management, and simplifies auditing processes for government agencies. Collaboration among relevant units is further promoted through the sharing of BIM data on the blockchain, advancing the application of BIM technology in operational management.

This study conducted two experimental scenarios based on a smart city BIM project to validate the effectiveness and performance of the proposed solution. Scenario one aimed to compare traditional blockchain registration with the proposed LB registration. We registered 30 virtual user accounts in both blockchain environments and conducted a detailed comparative analysis of the registration process efficiency and resource consumption. The practical advantages of the LB registration were assessed by comparing registration time metrics. Scenario two focused on evaluating the performance of the constructed blockchain network. Comprehensive tests were conducted on the network’s latency and throughput. The latency tests primarily assessed the time interval from transaction submission to confirmation to evaluate the network’s response speed and processing efficiency. The throughput tests measured the network’s transaction processing capacity within a given time unit, providing insights into the system’s overall handling capability. These tests comprehensively elucidated the performance of the constructed blockchain network under various load conditions, providing robust data support for its practical application in the management of operational information in smart city BIM projects.

### 4.3 Results and discussions

**Fig 9:**
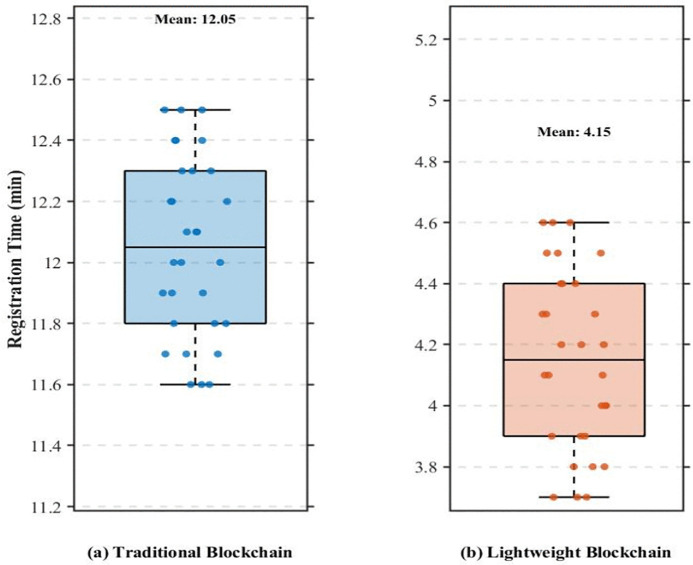
Comparison of user registration times between traditional blockchain and lightweight blockchain.

This study conducted a comparative analysis of registration times between traditional blockchain architectures and the proposed LB solution. To ensure the statistical validity of the performance evaluation, user registration testing was conducted for 30 rounds. Experimental findings revealed that the traditional blockchain registration process averaged 12.05 minutes, whereas the LB registration proposed herein averaged only 4.15 minutes, as depicted in Fig 9. These results demonstrate a significant enhancement in processing efficiency for BIM O&M information in smart cities through the proposed solution. The reduction in registration time facilitates faster user onboarding and data uploading, thereby enhancing the overall system’s response speed and operational efficiency. Such enhancements are crucial for the efficient management and real-time updating of BIM information in complex smart city environments.

**Fig 10:**
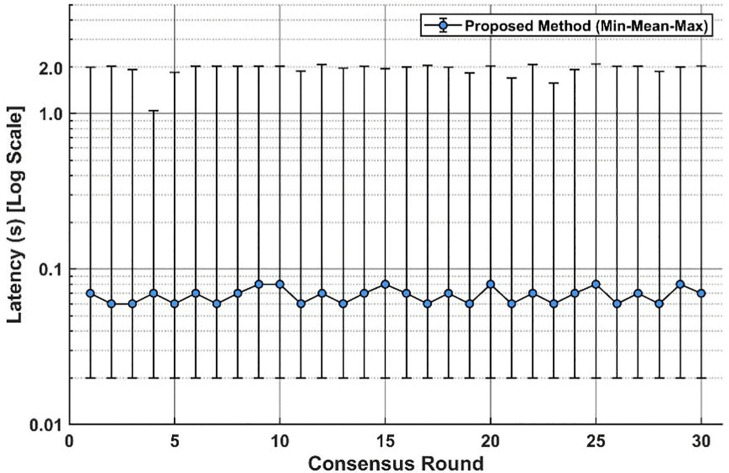
Blockchain latency.

According to the Hyperledger Caliper report, the benchmark workload includes a write/invoke operation (Set value, 5000 successful transactions, send rate 69.1 TPS, measured throughput 69.0 TPS) and a read/query operation (Query value, 26719 successful queries, send rate 921.2 TPS, measured throughput 921.1 TPS). The effective test duration for each workload can be derived from the Caliper summary (approximately 72.5 s for Set value and 29.0 s for Query value, computed as successful requests divided by measured throughput). Network latency and throughput serve as primary metrics for assessing the performance of a blockchain network, which are crucial for determining compliance with target requirements. Latency, specifically, denotes the transaction cost within the blockchain, a metric of paramount importance where high latency is deemed unacceptable for real-time construction coordination. In this broad-scale validation, thirty consensus rounds were conducted to evaluate system stability. As illustrated in Fig 10, the system achieved an average latency of approximately 68ms, with the error bars indicating the range between minimum and maximum latency values on a logarithmic scale. The findings underscore that achieving millisecond-level latency is imperative for smart contracts, and the proposed framework consistently maintains latencies within acceptable limits across all test rounds.

**Fig 11:**
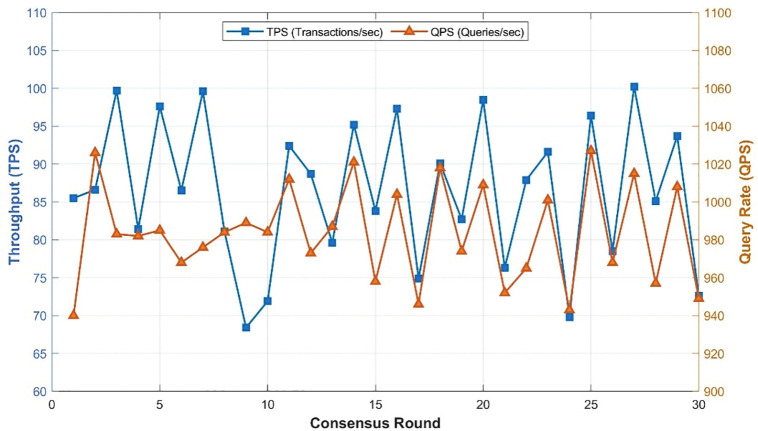
Blockchain throughput.

Throughput serves as a metric indicating the blockchain network’s transaction processing capability, quantified by the number of transactions per second (TPS) and queries per second (QPS). As depicted in Fig 11, the network achieves an average throughput of 86.5 TPS and 983.5 QPS over the 30-round consensus process (Table 1). Thus, it can be inferred that the blockchain network’s throughput adequately meets the majority of operational demands in large-scale engineering projects. Nonetheless, it is essential to note that both latency and throughput are subject to variations with changes in network configurations. Therefore, careful consideration of relevant computer configuration requirements is imperative during blockchain network deployment.

**Table 1:** Summary of network performance over 30 rounds (measured by Hyperledger Caliper).

Metric	Mean ± Std	Min–Max	Unit
Avg latency	68.0 ± 7.6	60–80	ms
Min latency	20.0 ± 0.0	20–20	ms
Max latency	1928.7 ± 201.7	1040–2080	ms
Throughput (TPS)	86.5 ± 9.6	68.4–100.2	tx/s
Throughput (QPS)	983.5 ± 26.0	940–1027	queries/s

The paper delineates its focus into two pivotal areas: one addresses strategies aimed at reducing the barriers to blockchain adoption, while the other tackles the complexities inherent in solving intricate BIM coordination issues, particularly those involving smart contracts. These dual strands of research hold significance across different domains, offering innovative solutions to real-world challenges. In the exploration of mitigating the hurdles to blockchain application, the research concentrates on proposing methodologies to streamline and enhance blockchain technology, facilitating its comprehension and practical implementation. This facet of the study not only fosters the widespread adoption of blockchain across commercial and social domains but also facilitates user-friendly digital transactions and asset management, thereby catalyzing the growth of the digital economy. On the other hand, the investigation into resolving intricate BIM coordination issues, notably those pertaining to smart contracts, addresses the imperative need for data consistency and collaboration efficacy during BIM’s design and construction phases. By introducing multi-type smart contract technology, the endeavor is geared towards establishing an efficient BIM coordination platform, facilitating automated identification, matching, and integration of building data. This, in turn, enhances collaboration efficiency among design and construction teams while minimizing errors and conflicts. Research endeavors in this sphere are poised to offer critical impetus to the construction industry’s digital transformation, elevating the quality and efficiency of construction projects and thereby fostering sustainable industry development.

Extensive comparative analysis highlights several significant advantages of the research plan outlined in this paper. Compared with representative BIM–blockchain approaches that typically rely on full-scale permissioned blockchains with per-user onboarding and generic contract designs, our framework is intentionally engineered for BIM O&M deployment by reducing onboarding overhead through the LB one-to-many mapping toolkit and aligning on-chain logic with practical collaboration via multi-type smart contracts (initialization–approval–query). The LB framework designed in this study markedly simplifies the registration process compared to traditional blockchain technologies, offering a more user-friendly interface. This enhancement facilitates a quicker understanding of blockchain technology and its applications in real-world scenarios, thereby improving work efficiency.

Moreover, the adoption of multi-type smart contract technology not only saves valuable time resources compared to traditional cloud-sharing platforms but also underscores the fundamental principles of immutability and decentralization inherent in blockchain technology. The inherent features of automatic execution and decentralized storage in multi-type smart contracts ensure data integrity and reliability, effectively reducing the risk of data tampering. Additionally, the decentralized architecture minimizes single points of failure, enhancing system stability and availability. The proposed solution demonstrates the core advantages of blockchain technology by simplifying the registration process and reducing time and cost, providing users with a more efficient, secure, and reliable blockchain application experience. These advantages not only facilitate the adoption and integration of blockchain technology but also offer robust support for the digital transformation and sustainable development of related industries.

## 5. Conclusions

This research endeavors to develop an innovative BIM O&M information management framework tailored for smart cities, leveraging LB and multi-type smart contracts to address prevailing challenges encountered in current BIM project O&M management. Through exhaustive research and practical implementation, three primary research objectives have been achieved, furnishing robust technical underpinnings for the establishment and advancement of smart cities. Distinct from prior BIM–blockchain studies that mainly present conceptual data-sharing architectures, we emphasize deployment-oriented mechanisms (lightweight onboarding and workflow-specific contracts) and validate them quantitatively in realistic BIM O&M scenarios. Firstly, this study pioneers the integration of LB into BIM O&M management, introducing a one-to-many mapping tool scheme to tackle the issue of high user mobility. This scheme facilitates the registration of multiple users under a single blockchain node, streamlining blockchain operations, trimming management overheads, and significantly enhancing user experience. Consequently, users can more seamlessly engage in operation and maintenance management, access requisite information in real-time, and facilitate efficient transmission and sharing of data. Moreover, the solution optimizes node resource utilization, bolstering system stability and efficacy. Secondly, a multi-type smart contract system tailored to the operational requisites of BIM project O&M management is developed. These smart contracts incorporate features such as data validation, permission management, and event triggering, offering configurable adaptability to diverse business needs. The automated execution of smart contracts yields notable enhancements in management efficiency, curtails the risk of human error, and augments the equity and transparency of management practices. Furthermore, the application of smart contracts mitigates management and time overheads, rendering the overall O&M management process more efficient and user-friendly. Lastly, the research framework’s feasibility was validated through a practical example, wherein tests revealed that the framework’s latency and throughput met existing operational demands, affirming its potential for real-world applications. These validation outcomes buttress subsequent research endeavors and establish a robust groundwork for advancing BIM O&M information management in smart cities.

Despite the contributions of this study, several limitations suggest clear avenues for future research. Firstly, the current framework focuses primarily on improving BIM data storage efficiency on a lightweight blockchain, and therefore does not include an in-depth security analysis regarding resistance to common blockchain attacks (e.g., Sybil attacks, double-spending, or node compromise). Future work will incorporate systematic security evaluation and explore enhanced protection mechanisms to strengthen the robustness of the lightweight architecture. Secondly, the current fingerprint-generation process still involves partial manual operations, which introduces additional workloads and potential inconsistencies. Future research will investigate AI-assisted automation to improve efficiency and reduce human intervention. Finally, considering the scale and heterogeneity of smart-city data, a single-chain blockchain architecture may face scalability constraints. Subsequent research will therefore explore cross-chain communication mechanisms and cloud-integrated deployment models to support larger-scale, distributed smart-city environments.
